# Does the combination use of two pain assessment tools have a synergistic effect?

**DOI:** 10.1186/s40560-016-0195-7

**Published:** 2017-01-03

**Authors:** Takeshi Suzuki

**Affiliations:** Department of Anesthesiology and General Intensive Care Unit, Keio University School of Medicine, 35 Shinanomachi, Shinjuku-ku, Tokyo, 160-8582 Japan

**Keywords:** Pain management, Critically ill patients, Pain assessment tools

## Abstract

Pain management is a very important aspect in the intensive care unit (ICU), as adequate pain control has been shown to be associated with better clinical outcomes in critically ill patients. A Numerical Rating Scale (NRS) ranging from 0 to 10 (0, no pain; 10, maximum pain), which is based on a patient’s self-report, is the gold standard for pain evaluation in patients who can communicate their pain intensity. On the other hand, it is very difficult to evaluate the degree of pain in critically ill patients owing to decreased consciousness level, delirium, and the effect of sedation for mechanical ventilation management. The Behavioral Pain Scale (BPS) and Critical Care Pain Observation Tool (CPOT) have been developed for pain assessment in patients who cannot self-report their pain intensity, and recent research has confirmed their efficacy in clinical trials. In the study by Paolo et al., published in this journal, they have demonstrated that discriminant and criterion validities of BPS and CPOT are good for the assessment of pain in mechanically ventilated critically ill patients. Besides, the authors have also shown that the combination use of these two tools is superior to the use of each tool individually. In this commentary, I would like to describe the importance and the difficulty of pain assessment in critically ill patients, discuss the validity and the reliability of the two major pain assessment tools, BPS and CPOT, and consider the future direction of pain assessment in the ICU.

## Background

The majority of critically ill patients in the intensive care unit (ICU) often experience pain during usual care, such as turning position, endotracheal suctioning, surgical wound care, and insertion or removal of drain and catheters during their ICU stay [1, 2]. Pain assessment is very important in critically ill patients, as adequate pain management is associated with better clinical outcomes [3, 4]. On the other hand, inadequate pain management has been shown to result in unfavorable events, including disturbed sleep, delirium, post-traumatic stress disorder, changes in immunological status, myocardial infarction, prolonged mechanical ventilation and ICU stay, and increased mortality rates [4, 5]. The Numerical Rating Scale (NRS) ranging from 0 to 10 (0, no pain; 10, maximum pain), which evaluates the degree of pain based on a patient’s self-report, is the gold standard tool for pain assessment in patients who can communicate the degree of pain [6]. However, it is very difficult to assess the degree of pain in the ICU, as critically ill patients cannot self-report their degree of pain owing to sedation usage for mechanical ventilation, delirium, and decreased levels of consciousness. Furthermore, vital signs, including blood pressure, heart rate, and respiratory rate, do not seem to be valid for pain assessment in critically ill patients, since these values are likely to fluctuate due to underlying diseases and vasopressor therapies [7]. Thus, pain evaluation in critically ill patients is quite challenging for attending physicians and nurses in the ICU.

For patients who cannot communicate their pain intensity, the Behavioral Pain Scale (BPS) and Critical Care Pain Observation Tool (CPOT) are recommended for pain assessment in an international guideline (pain, agitation, and delirium guideline (PAD guideline)) [8]. Although both tools have some limitations, such as inaccuracy when evaluating patients with brain injuries or delirium [9, 10], recent clinical studies have demonstrated that BPS and CPOT are superior to other pain assessment tools [11, 12]. This is based on the evaluation of a number of parameters including inter-rater reliability, internal consistency, discriminant validity, and criterion validity. However, few studies have compared the validity and the reliability of these pain assessment tools simultaneously in mechanically ventilated critically ill patients.

## Main text

In the study by Paolo et al., the authors compared BPS and CPOT with regard to the criterion validity and the discriminant validity and evaluated BPS and CPOT both individually and in combination, with regard to their sensitivity and specificity for detection of pain, in mechanically ventilated critically ill patients during usual nursing care including painful procedures. They have demonstrated that both tools have similar good criterion and discriminant validities, and the combination use of both tools might improve the efficiency for pain assessment, since the combination use resulted in better sensitivity and specificity, compared with the use of either tool alone. Furthermore, they have shown that facial expression has the strongest effect on pain assessment. Although this study might bring a new insight to pain assessment, I would like to present several issues that should be discussed in this study.

First, it is questionable whether the combination use of two pain assessment tools has a synergistic effect on the improvement of pain assessment, even though the combined use of both tools resulted in higher sensitivity than the use of either tool individually and higher specificity than the use of CPOT alone. Considering that BPS and CPOT include three common parameters, that is, facial expression, body movement (upper limb movement in BPS), and ventilator compliance, I wonder if only summing both scores reflects better pain assessment, compared with each scale alone. Regarding the discriminant validity and the criterion validity of the combination use of two tools, which was not evaluated in this study, the results of these evaluations would be expected to be better considering that the discriminant and criterion validities for each scale, BPS and CPOT, were good. Furthermore, the authors evaluated BPS and CPOT and the combination use of these tools regarding sensitivity and specificity in only conscious patients who could self-report their pain intensity. Since BPS and CPOT have been developed to evaluate pain intensity in unconscious patients who cannot communicate their pain levels effectively, the combination use of two tools should be evaluated in unconscious patients or patients with delirium. It is very surprising that there were no patients who developed delirium, even though critically ill patients were included and midazolam was used for sedation in this study. Second, the discriminant validity should be examined during both painful and non-painful procedures in the same population. If the values evaluated by pain assessment tools are increased by both painful and non-painful procedures, the validity and reliability are questionable. Thus, evaluation of the discriminant validity should be performed during morning care, including painful procedures, and evening care without painful stimulation, in this study. Finally, it is very important to evaluate the degree of pain intensity rather than to detect the presence or absence of pain and prescribe analgesics according to each patient’s degree of pain without adverse events. This study did not examine if the combination use of two pain assessment tools can evaluate the degree of pain better than either scale alone. Further study is warranted to examine whether the combination use of two pain assessment tools can evaluate the degree of pain intensity accurately.

## Conclusion

Appropriate pain management is a very important aspect when caring for critically ill patients in ICUs. However, pain assessment in patients who cannot communicate their pain levels due to unconsciousness or delirium imposes challenges on attending physicians and nurses. Among many pain assessment tools, BPS and CPOT are recommended for the evaluation of pain intensity in critically ill patients. Although some review articles have reported certain limitations for both pain assessment tools [9, 10], the validity and reliability of BPS and CPOT have been confirmed in recent clinical studies [12, 13]. To improve the quality of patient care in ICUs, attending physicians and nurses have to consider how they can utilize these pain assessment tools efficiently to relieve critically ill patients from stressful pain stimulants. Further research should investigate whether appropriate pain management using these pain assessment tools can improve the prognosis of critically ill patients.

## Abbreviations

BPS: Behavioral Pain Scale
CPOT: Critical Care Pain Observation Tool
ICU: Intensive care unit
NRS: Numerical Rating Scale
PAD guideline: Pain, agitation, and delirium guideline
